# Communicative Behaviors in an Internet-Based Intervention for Individuals With Autism: Mixed Methods Analysis

**DOI:** 10.2196/76527

**Published:** 2026-02-04

**Authors:** Britta Westerberg, Karin Jacobson, Maria Unenge Hallerbäck, Susanne Bejerot, Fredrik Holländare

**Affiliations:** 1 School of Medical Sciences, Faculty of Medicine and Health Örebro University Örebro Sweden; 2 University Health Care Research Center Faculty of Medicine and Health Örebro University Örebro Sweden; 3 Department of Public Health Sciences Karlstad University Karlstad Sweden; 4 Centre for Clinical Research Region Värmland Karlstad Sweden

**Keywords:** autism, communicative behaviors, internet-based treatment, narrative identity

## Abstract

**Background:**

To meet the needs of individuals diagnosed with autism, internet-based interventions have been developed with a variety of objectives. A deeper understanding of the mechanisms of change may help tailor interventions to individual needs. The communicative behaviors of individuals with autism participating in text-based internet-based interventions remain largely unexplored, as do their potential relations to clinical outcomes. An improved understanding of participants’ behaviors may help therapists better tailor support, promote engagement, and enhance treatment outcomes.

**Objective:**

This study aimed to explore the communicative behaviors of individuals with autism participating in an internet-based intervention and to examine whether different behavioral patterns were associated with treatment outcomes or treatment adherence.

**Methods:**

Messages from 34 participants enrolled in an 18-week internet-based cognitive behavioral therapy program were analyzed using abductive qualitative content analysis. Correlational analyses were used to examine the relationships between qualitative categories and change scores on outcome measures and rates of module completion.

**Results:**

Fourteen behavioral categories were identified and grouped into three overarching domains: (1) “This is me,” which encompasses the participants’ narratives on identity, personality, autistic functioning, current and past circumstances, and worldview; (2) “Working with the treatment,” which included statements related to engagement with the treatment process; and (3) “I struggle,” which comprised of past and present negative experiences and challenges. Correlational analyses revealed associations between several behavioral categories and improvements in quality of life and treatment adherence.

**Conclusions:**

The findings highlight the importance of self-narrative formulation among individuals with autism and suggest that certain communicative behaviors—particularly those involving identity reflection and recognition of treatment-related gains—were positively associated with therapeutic outcomes. The findings enhance our understanding of how individuals with autism engage in internet-based cognitive behavioral therapy and may serve as a valuable source of information for therapists when guiding expectations regarding client outcomes and identifying participants who may benefit from additional support.

**Trial Registration:**

ClinicalTrials.gov NCT03570372; https://clinicaltrials.gov/study/NCT03570372

## Introduction

### Background

Autism is a neurodevelopmental condition characterized by difficulties in social communication and a stereotypical behavioral pattern [1]. These difficulties imply functional impairment in social situations and may affect psychological well-being [2,3] and low quality of life (QOL) [4]. Furthermore, individuals with autism face several challenges in accessing health care [5], and internet-based interventions offer a convenient and flexible format that may increase accessibility for this population.

The evidence base for internet-based interventions is growing, and an increasing number of diagnostic groups are being offered internet-based cognitive behavioral therapy (ICBT) [6]. Given the efficacy of ICBT, there is a need for an improved understanding of the mechanisms of change, as this knowledge would allow more targeted adaptations of treatment content to the specific needs of each service user [7].

Interventions that include therapist support tend to be more effective than those delivered without guidance [8]. In addition, factors such as participant adherence, treatment credibility, working alliance, and baseline symptom scores have all been identified as important predictors of treatment outcome in ICBT [9]. In particular, therapeutic alliance and treatment adherence are related to favorable outcomes [10,11].

Therapeutic feedback in ICBT is typically provided at regular intervals [7], in response to participant progress, and the participant may have the possibility to write to the therapist at their convenience, both in response to therapist feedback and to initiate a new conversation. Though the core ingredients in ICBT are expected to be embedded within the predetermined text-based modules or sessions, the established importance of therapeutic support [8] and alliance [10] implies that some change mechanisms are influenced by the one-to-one written communication.

The functional components of therapist feedback in ICBT, conceptualized as “therapist behaviors,” are important for both adherence and outcome [12-14]. The client-related equivalent—patient behaviors in ICBT—remains less thoroughly explored. However, previous research indicates that different communicative patterns among participants in ICBT may also be associated with both outcome and adherence [15-17]. Increased insight into what is communicated by clients in ICBT is an important piece of the puzzle in forming an understanding of who is likely to benefit from the program—and who may not—as well as identifying individuals at risk of dropping out. This information can also serve as a valuable indicator to practitioners that a participant is struggling and may require additional support [15].

Svartvatten et al [11] explored behaviors reflected through client messages in ICBT for depression, and their relation to outcome and adherence. They identified 10 behavioral categories and found that “Alliance and Observes positive consequences” correlated positively with changes in outcome, and the behaviors “Alliance,” “Identifies patterns and problem behaviors,” “Maladaptive repetitive thinking,” “Observes positive consequences,” “Tries alternative behavior,” “Chooses alternative behavior,” and “Avoidance of treatment” were positively related to the number of modules completed.

In 2 comparative studies, Soucy et al [18] and Kraepelien et al [19] adopted the behavioral categories from Svartvatten’s study to examine whether these could be generalized to their own data, including participants with depression, anxiety [18], and alcohol use disorder [19]. Although both studies found the predefined categories applicable, notable differences in both frequency and relation to adherence and outcome were observed. Based on inconsistent correlation results, both studies refrained from concluding the predictive value of client behaviors but suggested that the categories may still offer insight into the therapeutic process in ICBT. Soucy et al [18] noted that their directed (also referred to as deductive) content analysis approach—following the categories generated by Svartvatten—may have hindered the detection of other relevant themes. Therefore, they call for future research to analyze client messages inductively, without referring to pre-existing frameworks.

An inductive analytical approach may be especially justified when analyzing communicative patterns among individuals with autism, a population known to exhibit a qualitatively different communicative style compared to the norm, both generally [20] and in text-based online communication [21]. These communication differences may be manifested, for example, by an impaired narrative ability—a communicational skill important for sharing experiences and connecting with others [22]. Furthermore, research has shown that computer-mediated communication often is preferred over verbal communication among individuals with autism [21,23] and that they use internet-based communication in qualitatively different ways than individuals without autism [21,24,25]. These findings open up the possibility that the communicative behaviors in ICBT for autism may differ from those of other patient groups, which justifies approaching this question with an inductive approach, unrestricted by the deductive categories used in previous studies.

To manage the increasing number of individuals diagnosed with autism and to meet their needs, internet-based interventions have been developed with a variety of objectives, including increasing QOL [26], providing psychoeducation [27], and treating psychiatric comorbidities [28-30]. Although the research base on ICBT for autism is relatively scarce, the available studies suggest that internet-based interventions may be both particularly suitable for the needs of individuals with autism [27,31], as well as effective [27,29,30].

Within the current project, an 18-week internet-based intervention adapted for individuals with autism was evaluated for feasibility and effect compared to an active control group [26]. The results were complex and ambiguous; while no group-level effects were found on quantitative outcomes, participant satisfaction was high, and dropout rates were low, indicating good feasibility. A qualitative study on participant experiences of the intervention [31] showed that the participants generally appreciated the internet-based format, and especially the opportunity to communicate with the therapist in writing.

However, the participants had varying experiences regarding therapist support and the messaging function. Some participants used the messaging function to enable a deeper conversation with the therapist, whereas others only occasionally responded to the therapeutic feedback, resulting in great variability in the amount of therapeutic contact [31]. Accordingly, the level of support was, to a great degree, dependent on the behavior of the participants. Whether this diverse use of the therapeutic support may be related to the treatment effect or adherence is, however, unclear and warrants further investigation. As the active ingredients in ICBT for individuals with autism are yet unexplored, we should remain open to the possibility that the varying communicative behaviors of participants may play a role, even in interventions where no group-level effect was found.

Little is known about the behavior reflected through participant messages in internet-based interventions for individuals with autism, or how variations in these behaviors may relate to treatment outcomes and adherence. Analyzing participant messages in ICBT may offer valuable insights into how individuals with autism engage in the treatment. Furthermore, early identification of specific participant behaviors may inform professionals about what to expect when treating individuals with autism, and thereby guide therapeutic decision-making. Improved understanding of client behaviors could thus be helpful both in identifying individuals at risk of dropping out and in guiding the development of future programs.

### Objectives

This study aimed to gain knowledge about participants’ communicative behaviors in an internet-based intervention for individuals with autism by investigating the content of participant messages. An additional aim was to explore whether the use of different behaviors (including word count and message frequency) was related to clinical outcome or adherence.

## Methods

### Design

This study used a mixed method design, using data collected as part of a larger randomized controlled trial (RCT) aimed at evaluating an internet-based cognitive behavioral intervention to improve QOL in autistic adults (ClinicalTrials.gov NCT03570372). A qualitative analysis of participant text messages and the calculation of change scores were first conducted independently, after which the qualitative and quantitative data were integrated through correlation analyses. The integration allowed the exploration of how the qualitative categories were related to quantitative changes.

### Participants and Recruitment

All messages from participants in the intervention group (n=42) of an RCT on ICBT for individuals with autism [26] were included in the current qualitative analysis. The RCT was announced through posters in waiting rooms of health care facilities in Örebro county, Sweden, and as advertisements in the local press and on a social media platform (Facebook, Meta Platforms, Inc). Participants for the RCT were recruited by completing a digital self-application form administered through 1177, a Swedish national platform for online health care. The application form included questions on age, gender, living arrangement, occupation, and age at diagnosis. The inclusion process involved a structured interview based on the Mini International Neuropsychiatric Interview, along with a screening questionnaire covering autistic symptoms. Diagnoses were confirmed by collecting assessment records or by verbal confirmation from clinicians. A detailed description of the recruitment process and inclusion criteria of the RCT is provided in Westerberg et al [26].

### Intervention

The intervention was based on an evidence-based cognitive behavioral therapy group treatment for adults with autism [32,33], which had been further developed, condensed, and adapted to an internet-based format by the first author, BW.

The intervention lasted 18 weeks. The intervention aimed to enhance QOL and sense of coherence (SOC) and to decrease psychiatric symptoms through the completion of 18 text-based modules focusing on themes relevant to these objectives (refer to Table S1 in Multimedia Appendix 1 for an overview of the themes). Each module included psychoeducation, exercises, and strategies based on cognitive behavioral therapy to enhance coping with everyday life challenges, but adapted to the needs of individuals with autism. In line with recommendations for such adaptations [34], a significant portion of the program was dedicated to psychoeducational content about autism, individual variations, and common comorbidities. The intervention also introduced tools and concepts to support self-understanding and provided terminology to describe participants’ functioning. Every 2 weeks, the participants were invited to take part in a live chat session together with other participants, focused on discussing the theme of the most recent module.

In most modules, home exercises were designed to be completed either using text-based worksheets or as “field work” to be reported on the online platform. All modules also included reflective questions to be answered directly in conjunction with the text. The documentation and reports from the exercises, along with responses to the reflective questions, formed the basis for the therapeutic feedback.

Therapeutic feedback was delivered asynchronously via a messaging function available on the same platform as the treatment. The messaging function resembled an email page, allowing participants to respond to therapeutic feedback and initiate new conversations. Therapists were expected to reply within 1 working day. However, participants were not required to send any messages via this function.

### Material

All messages written by participants using the messaging function were included. Aside from the removal of information that may reveal participant identities (such as names, telephone numbers, or web addresses), the messages were not modified in any way.

Change scores (from baseline to posttreatment) on outcomes from the RCT were included for a quantitative correlation analysis. The Brunnsviken Brief Quality of Life Scale (BBQ) [35], consisting of 12 items (responses ranging from 0=do not agree to 4=totally agree), was used to assess QOL. Each item related to satisfaction in a specific life area was weighted against a rating of the importance of that area. The multiplied products of each item-pair (satisfaction × importance across 6 life areas) were summed to obtain a total QOL score (range: 0-96), with higher scores representing a higher QOL.

The 13-item Sense of Coherence (SOC-13) scale was used to assess the concept of SOC [36]. SOC reflects the extent to which an individual perceives life events as coherent and comprehensible and life demands as manageable and meaningful. Items are rated on a 7-point scale, with a higher total score (range: 13-91) indicating a stronger SOC.

To assess symptoms of depression and anxiety during the past week, the Hospital Anxiety and Depression Scale (HADS) for depression (HADS-D; 7 items) and anxiety (HADS-A; 7 items) subscales were used [37]. Items are rated on a 4-point scale, with higher scores (range: 0-21) indicating greater symptom severity.

Word count and the frequency of participant and therapist messages were analyzed in relation to the outcome. Generic messages sent to all participants (such as reminders of the peer participant chat sessions) were removed from the data at an early stage. Similarly, messages whose sole purpose was to inform the participant that they had received a new module or to remind them to complete self-assessments were excluded from the analyses.

### Qualitative Analysis

The material was analyzed using both a directed and conventional approach to content analysis [38], adopting a combination of inductive and deductive perspectives, resulting in an abductive analytical process [39]. All coding and sorting were conducted using NVivo qualitative data analysis software (Lumivero, LLC) [40] by 2 licensed psychologists (BW and KJ). BW was also 1 of 4 therapists during the trial and was therefore familiar with both the foundational elements and components of the program, as well as the specific setting and design of the trial. As personal identifiers were removed from the texts before analysis, the participants’ identities were not revealed through the texts. However, the coding process was not fully blinded, as—due to her previous role as a therapist—BW could recognize certain utterances as stemming from individual participants.

Initially, all messages were read to gain an overall sense of the material and its character. Thereafter, BW segmented and condensed the texts into meaning units, after which BW and KJ jointly and inductively coded the meaning units from 5 participants and discussed the codes to develop a preliminary coding framework. All utterances that could be considered reflective of a behavior were given a descriptive code to capture the manifest content of the participant’s messages.

In this analysis, a behavior was defined as statements in the participant messages that appeared to serve a function or convey an intention, either in relation to internal processes or to the treatment work or the therapist. These included both utterances with semantic and meta-linguistic content. Statements that—although functional—were considered not to be of importance for the research questions, such as using polite phrases (“have a good day”) or clarifying the structure of the messages (“firstly I will answer your questions”), were excluded.

When a preliminary coding framework was established, the 2 researchers independently coded the texts of 1 participant and compared their interpretations and the wording of codes to refine the codes and to reach consensus regarding the final framework to be used thereafter. Once agreement regarding the wording of codes was achieved, BW coded the communication from the remaining 28 participants in the same manner.

When the texts of approximately 10 individuals had been coded, BW began inductively sorting the codes into clusters illustrating similar behaviors. During this process, a pattern emerged in which several of the clusters resembled categories identified in earlier research [11,18,19]. A decision was made to allow the use of previously identified categories [11] when a category was identified that clearly aligned with one of these. This resulted in an abductive approach, meaning that we moved iteratively between the data and the categories identified in earlier research, which were refined and adjusted to better fit our data during this process. Given the large amount of data and codes, coding and sorting were conducted alternately until all relevant meaning units had been coded and sorted. No third-party validation was conducted during the analysis process.

### Quantitative Analysis

The frequencies of each participant’s contribution of codes to the categories derived from the qualitative analysis were used in correlation analyses to explore relationships with the quantitative change scores. Change scores on BBQ, SOC-13, HADS-D, and HADS-A were calculated by subtracting preintervention scores from postintervention scores. Normality of the variables was assessed using the Shapiro-Wilk test.

To assess behavior categories in relation to module completion independently of the amount of text written, the individual frequency of codes in each category was divided by the total number of words written by each participant. Accordingly, the frequency of each behavior category was calculated as a proportion of the total number of words, which minimized the influence of overall text length (which would be strongly dependent on the number of modules completed), as the aim was to examine the relative prevalence of behaviors rather than general writing activity.

BBQ, HADS-D, and HADS-A were all normally distributed. However, as the distribution of SOC-13, all behavior category variables, word and message frequency, and module completion rates deviated from normality, Spearman rank-order correlation (ρ) was used to assess the associations between behavior categories and change scores, module completion, and word and message frequency. No adjustment for multiple testing was made, as the study was exploratory and did not involve predefined statistical hypotheses. All quantitative analyses were performed using SPSS Statistics (version 29) [41].

### Ethical Considerations

The original trial received ethical approval from the Regional Ethics Committee in Uppsala, Sweden (ref no 2017/392), and the Swedish Ethical Review Authority later approved an amendment (ref no 2022-05792-02) with clarifications regarding this mixed methods study. At inclusion, all participants provided consent for their personal and medical data to be used for research purposes. All data were deidentified before analysis. Personal identifiers were removed from the analyzed text material, participants were assigned unique IDs, and the code key was accessible only to the research team. Participants did not receive any financial compensation for participation in this study. However, a gift card of 300 SEK (**≈** US $32) was provided to participants on completion of the postintervention and follow-up assessments of the original RCT.

## Results

### Overview

As 8 participants did not provide any written material, the qualitative analysis included messages from 34 participants. Table 1 provides the baseline characteristics of the participants and the number of completed modules.

**Table 1:** Baseline characteristics and adherence of the participants included in the qualitative analysis (N=34).

Characteristic		Value
Age (years), mean (SD)		33.8 (10)
**Gender, n (%)**		
	Men	14 (41.2)
	Women	18 (52.9)
	Other (nonbinary or transgender)	2 (5.9)
**Habitation, n (%)**		
	With partner and/or children	12 (35.2)
	With parents	9 (26.5)
	Alone	11 (32.4)
	Group or serviced housing	1 (2.9)
	Other	1 (2.9)
**Age when diagnosed (years), n (%)**		
	<19	11 (32.4)
	20-35	14 (41.2)
	>36	9 (26.5)
**Occupation, n (%)**		
	Employed	4 (11.8)
	Daily activities	2 (5.9)
	Student	4 (11.8)
	Job seeker	9 (26.5)
	Sick leave	7 (20.6)
	Other	8 (23.5)
Completed modules, mean (SD) and median (IQR)		15.6 (4.2) and 18 (14.8-18)

### Categories From the Qualitative Content Analysis

In total, 2569 codes were identified, of which 2476 were considered relevant to the aim. These were sorted into 14 categories, which were further grouped into 3 overarching domains: “This is me” (1092 codes and 4 categories), “Working with the treatment” (861 codes and 6 categories), and “I struggle” (523 codes and 4 categories). Table 2 provides an overview and the relative frequency of codes (%) across domains and categories, as well as definitions of each category. Refer to Table S2 in Multimedia Appendix 1 for example codes and quotes from each category.

**Table 2:** Overview of domains, categories, and the relative frequency of codes (%), and definitions of each category.

Domain and category, %			Definition
**This is me, 44.1%**			
	This is who I am, 15.5%	Texts reflecting a personal narrative about what kind of person one is, what qualities, difficulties, and needs they have, their autistic functioning, and how they (think that they) are perceived by others.	
	My present and past circumstances, 8.2%	Information on current events, everyday life, positive experiences, and early formative events.	
	Change is possible, 14.2%	Strategies that work or have potential to work, that they have developed through life, and show motivation and insight that they have the agency to act to progress.	
	My point of view, 6.2%	Beliefs, opinions, and thoughts about society, autism, human psychology, and the perspective of others.	
**Working with the treatment, 34.8%**			
	Appreciation and treatment alliance, 8.4%	Expressions of appreciation, a positive attitude, and bonds of alliance toward the treatment or the therapist.	
	Putting the treatment aside, 3.2%	Information about not having completed parts of the treatment, and reasons for this, including both psychological issues, such as forgetting and lacking energy, but also external events that got in the way of the treatment work.	
	Plans to attempt a new task, 7.0%	Reflections regarding how to – or plans to implement a treatment task or exercise, or deciding on a treatment goal.	
	Have attempted a new task, 4.6%	Reports on completion of a treatment task or exercise, or reflections around the implementation of a task or exercise.	
	Observing positive consequences of treatment, 3.2%	Statements illustrating that the participant has observed a positive consequence on their personal development from the treatment or specific exercises.	
	Problems with the treatment, 8.3%	Texts in which the participant expresses difficulties, frustration, or other negative aspects of the treatment content or format, such as failure to understand a task or its purpose, considering certain parts of the treatment irrelevant, or technical problems.	
**I struggle, 21.1%**			
	Life is and has been demanding, 11.6%	Descriptions of current and past difficult events or circumstances, that they have been treated badly throughout life, experienced failure, and have been enduring from mental ill-health.	
	Identifies patterns and problem behaviors, 3.1%	Texts reflecting identification and insight into maladaptive and safety behaviors, and identification of the relation between these behaviors and negative consequences.	
	I am troubled by mental ill-health, 4.3%	Factual descriptions of current mental ill-health, loneliness, and struggles and their causes and consequences, without engaging in maladaptive thoughts or rumination.	
	Maladaptive thoughts, 2.2%	Expressions of hopelessness, meaninglessness, or distress, as well as other cognitive distortions, indicate a stagnation of cognitive flexibility.	

The domain “This is me” consists of the categories “This is who I am,” “My present and past circumstances,” “Change is possible,” and “My point of view.” The domain covers participants’ descriptions of themselves in terms of personality, abilities, and difficulties, as well as current and past life situations that have influenced who they are. It further comprises their beliefs and opinions, their view of life and others, how they have used strategies to manage problems, and how they have developed, or are motivated to develop and function better. This domain illustrates that participants have insight into their agency, responsibility, and ability to influence their situation, but also reflects self-awareness regarding how their autistic difficulties pose challenges in this.

The domain “Working with the treatment” contains the categories “Appreciation and treatment alliance,” “Putting the treatment aside,” “Plans to attempt a new task,” “Have attempted a new task,” “Observing positive consequences of treatment,” and “Problems with the treatment.” These categories include statements that are in some way related to participants’ engagement in the treatment. It involves both positive and negative experiences and effects of the treatment, as well as reports on completed tasks and plans for future tasks. Reasons why certain parts of the treatment were not carried out are also included in this domain.

The domain “I struggle” contains the categories “Life is and has been demanding,” “Identifies patterns and problem behaviors,” “I am troubled by mental ill-health,” and “Maladaptive thinking.” This domain contains descriptions of past and current negative events, experiences of failure, descriptions of a difficult life situation, experiences of mistreatment, and how these have negatively affected mental health. It also covers how their mental ill-health manifests in symptoms and negative behavioral patterns.

Nine of the categories were found to correspond to categories identified in previous studies. Refer to Table S3 in Multimedia Appendix 1 for an overview of the categories from this study and—where applicable—the corresponding category from the studies by Svartvatten et al [11] and Kraepelien et al [19]. Five categories not previously described by the literature were identified in this study. These are: “This is who I am,” “My present and past circumstances,” “Change is possible,” “My point of view, and “Life is and has been demanding.” Two categories identified in previous research, “Confrontational alliance rupture” [11,18,19] and “Observes alcohol-related setback” [19], were not identified in our data.

### Correlation Between Frequency of Behaviors and Outcome

Table 3 provides baseline, posttreatment, and change scores on outcomes, and Table 4 provides correlations between the frequency of participant behaviors and change scores of outcomes and the number of completed modules. As 5 participants—although included in the qualitative analysis—did not complete the postassessment, the correlational analyses include data from 29 participants.

The domains “This is me” and “Working with the treatment” and the categories “This is who I am,” “Change is possible,” “My point of view,” “Appreciation and treatment alliance,” “Plans to attempt a new task,” “Have attempted a new task,” and “Observing positive consequences of treatment” correlated significantly with a positive change in BBQ. The domain “This is me” and the categories “This is who I am,” “Change is possible,” and “Identifies patterns and problem behaviors” correlated significantly with a higher number of completed modules. No other significant correlations between the frequency of behaviors and outcomes were observed.

These findings show that when participants more frequently expressed aspects of self-understanding (as in the domain “This is me”) and engaged in behaviors related to the treatment (as in the domain “Working with the treatment”), they tended to experience greater improvements in QOL (BBQ) and complete more treatment modules. This pattern indicates that behaviors reflecting active engagement and self-reflection may play an important role in facilitating positive treatment outcomes.

A total of 515 participant messages were sent. The median number of words per participant was 825 (IQR 530-2110; range 64-5562), and the median number of messages per participant was 11 (IQR 5-18; range 1-75). There was a significant moderate positive correlation between change in BBQ score and the number of participant words (ρ=0.52; *P*=<.001) and the number of participant messages (ρ=0.42; *P*=.02). There was no significant correlation between the number of words written by the therapist and any of the outcome variables.

**Table 3:** Means and distribution of pre- and posttreatment scores, change scores, and paired samples t test results on outcomes of the participants included in the quantitative analysis (N=29).

Outcome	Pre, mean (SD); range	Post, mean (SD); range	Change score^a^, mean (SD)	*t* test (*df*)	*P* value
BBQ^b^	41.14 (21.82); 0-96	45.90 (24.19); 0-96	4.76 (14.46)	–1.77 (28)	.09
SOC-13^c^	48.17 (15.01); 22-90	49.07 (14.49); 21-85	0.90 (9.82)	–0.49 (28)	.63
HADS-D^d^	7.45 (4.03); 0-15	6.83 (5.34); 0-19	–0.62 (3.63)	0.92 (28)	.37
HADS-A^d^	12.31 (4.97); 2-20	11.45 (5.10); 1-21	–0.86 (3.0)	1.55 (28)	.13

^a^Change scores represent posttreatment minus baseline values.

^b^BBQ: Brunnsviken Brief Quality of Life Scale.

^c^SOC-13: 13-item Sense of Coherence scale.

^d^HADS-D and HADS-A: Hospital Anxiety and Depression Scale for depression and anxiety subscales.

**Table 4:** Correlations (Spearman ρ) between relative frequency of participant behavior and outcome change-scores and module completion (N=29).

Domain and behavioral category		BBQ^a^		SOC-13^b^		HADS-D^c^		HADS-A^c^		Module completed^d^	
		ρ	*P* value	Ρ	*P* value	ρ	*P* value	ρ	*P* value	ρ	*P* value
**This is me**		0.51	<.001	0.04	.83	–0.21	.27	–0.05	.80	0.45	.01
	This is who I am	0.50	.01	0.08	.69	–0.19	.31	–0.02	.91	0.43	.01
	My present and past circumstances	0.32	.09	0.00	1.0	–0.21	.27	–0.08	.67	0.21	.24
	Change is possible	0.39	.04	0.09	.65	–0.20	.30	0.02	.93	0.37	.03
	My point of view	0.53	<.001	0.21	.27	–0.14	.48	–0.04	.84	0.16	.36
**Working with the treatment**		0.50	.01	0.05	.82	–0.25	.20	0.02	.92	–0.11	.55
	Appreciation and treatment alliance	0.44	.02	–0.06	.77	–0.11	.57	0.07	.74	–0.08	.65
	Putting treatment aside	0.25	.19	–0.13	.49	–0.34	.07	–0.09	.64	–0.09	.62
	Plans to attempt a new task	0.39	.04	–0.02	.92	–0.19	.31	0.12	.53	–0.00	.99
	Have attempted a new task	0.41	.03	–0.06	.77	–0.23	.23	–0.03	.89	0.09	.63
	Observing positive consequences	0.50	.01	0.12	.53	–0.22	.24	–0.03	.86	0.30	.08
	Problems with the treatment	0.27	.16	0.02	.90	–0.14	.46	0.08	.67	0.18	.31
**I struggle**		0.31	.10	0.03	.88	0.05	.79	0.20	.31	–0.06	.75
	Life is and has been demanding	0.32	.09	–0.10	.62	–0.04	.83	0.12	.53	–0.15	.40
	Identifies patterns and problem behaviors	0.31	.11	0.12	.55	–0.08	.68	0.00	1.0	0.36	.03
	I am troubled by mental ill-health	0.11	.56	–0.00	.99	0.07	.71	0.26	.17	0.32	.06
	Maladaptive thoughts	0.14	.47	0.31	.10	0.26	.18	0.20	.30	–0.04	.82

^a^BBQ: Brunnsviken Brief Quality of Life Scale.

^b^SOC-13: 13-item Sense of Coherence scale.

^c^HADS-D and HADS-A: Hospital Anxiety and Depression Scale for depression and anxiety subscales.

^d^Controlled for the total number of words.

## Discussion

### Principal Findings

The purpose of this study was to explore written communicative behaviors among individuals with autism participating in an internet-based intervention aimed at improving QOL and to examine whether any of these behaviors were associated with treatment outcomes.

The results show that statements related to participants’ descriptions of themselves in terms of personality, abilities, beliefs, experiences, and personal development were highly prevalent, with nearly half of the codes (44.1%) categorized under the domain “This is me.” The most common category was “This is who I am” (15.5%), which included reflections on personality and autistic functioning. The second most frequent category was “Change is possible” (14.2%), consisting of statements expressing awareness that personal development and growth are possible, such as narratives of past growth or coping strategies perceived as helpful. “Life is and has been demanding,” also accounting for a substantial portion (11.6%) of the codes, captured descriptions of adversity, experiences of mistreatment, failure, and mental ill-health.

Correlation analyses revealed that the domains “This is me” and “Working with the treatment,” as well as the categories “My point of view,” “This is who I am,” and “Observing positive consequences of treatment,” were significantly moderately associated with improvements in BBQ scores. These findings suggest that the opportunity to reflect openly on identity, personal perspectives, and the perceived impact of treatment may be particularly important for improving QOL in individuals with autism. Notably, the finding regarding “Observing positive consequences of treatment” aligns with previous findings by Soucy et al [18] and Svartvatten et al [11], who also reported a positive correlation between this behavior and treatment outcomes.

The significant moderate positive relation between BBQ scores and the category “Appreciation and treatment alliance” further supports a well-established body of research highlighting the importance of therapeutic alliance for the therapeutic outcomes [9-11,42,43]. In addition, the category “Have attempted a new task” was moderately positively correlated with changes in BBQ, whereas “Change is possible” and “Plans to attempt a new task” showed weak associations. These findings indicate that motivational expressions related to trying new strategies and behaviors—both within and beyond the treatment context—may reflect treatment engagement and efficacy.

Moreover, the number of words and messages written by participants was also positively associated with improvement in BBQ scores. While earlier studies examining text quantity have primarily focused on its relation to adherence—showing that more words are associated with increased program completion [17,44]—our results indicate that text quantity may also be related to changes in outcomes in ICBT for individuals with autism.

Interestingly, while several communicative behaviors were associated with improvements in QOL (as measured by BBQ), no associations were observed with changes in anxiety or depression (HADS) or sense of coherence (SOC-13). One possible explanation is that QOL, as a subjective and existential construct, may be more readily influenced by participants’ self-reflective and narrative expressions. In contrast, clinical symptoms of anxiety and depression, and the more stable SOC, may be less susceptible to short-term fluctuations driven by communicative behaviors. Despite earlier findings suggesting that SOC may improve from interventions [45]—which motivated its use as an outcome in the original RCT—this effect was not found in our study [26]. Another potential explanation is that the relatively greater pre-post variability in BBQ, compared with the smaller mean changes observed in HADS and SOC-13 scores, may have increased the likelihood of detecting statistically significant correlations for QOL but not for the other measures.

Regarding adherence, the domain “This is me” and the category “This is who I am” were moderately associated with the number of completed modules, while the categories “Change is possible” and “Identifies patterns and problem behaviors” showed weak associations. This partly supports previous findings by both Soucy et al [18] and Svartvatten et al [11], who also reported a positive correlation between “Identifies patterns and problem behaviors” and the number of modules completed.

Although no causal inferences can be made, the observed associations in this study may shed light on potential behavioral indicators that are beneficial for individuals with autism to engage in during internet-based psychological interventions.

The relatively large number of identified categories (n=14) illustrates a broad range of content shared by participants with their therapists and likely reflects the wide diversity among individuals with autism. Unlike ICBT, which targets specific disorders, this intervention focused on the individual’s whole life, and given the comprehensive scope of the treatment (refer to Table S1 in Multimedia Appendix 1), the extensive result is not surprising. Furthermore, an obvious reason for the relatively high prevalence of specific behaviors (ie, those reflected in the domain “This is me” and the categories “This is who I am” and “Change is possible”) is related to the treatment content itself, in which reflections on the self in relation to past, present, and future experiences were repeatedly encouraged.

Consequently, the content of this treatment may partly explain why the behavior categories in the current analysis were only partially similar to those identified in earlier studies [11,18,19]. The 4 categories constituting the domain “This is me,” and the category “Life is and has been demanding,” including meaning units not covered by earlier frameworks (Table S2 in Multimedia Appendix 1). In agreement with Soucy et al [18], our study supports the notion that a fully deductive process—that is, following the existing coding framework—would have hindered the detection of relevant themes.

Beyond treatment content, these divergent findings (compared with previous studies) must be considered in light of the unique characteristics of the study population—individuals with autism. A considerable proportion of the participant messages centered around identity, functioning, and experiences in relation to autism or psychiatric comorbidity, which were manifested in several ways. Some statements offered nonvaluing descriptions of how their autism contributed to shaping their identity, and others accounted for experiences and traits as contributing to personality formation independent of autistic traits. Regardless of etiology, it appeared fundamental for the participants to construct a narrative around who they are and how their functioning affects their lives. This internalized self-narrative—defining who they are, how they came to be, and where their life may be heading—can be described as their narrative identity [46,47].

Through the construction of a narrative identity, people define and communicate who they are to themselves and others [47,48]. This process fosters self-insight and understanding, allowing negative experiences to be reframed as opportunities for growth, which in turn has been linked to greater well-being [49,50]. Given the precondition of atypical functioning, the process of constructing a coherent self-narrative thus appears particularly relevant for individuals with autism. While research suggests that narrative skills [22,51] and identity formation [52] may be impaired in autism, Samra [53] highlights the importance of engaging with one’s narrative identity. In their thesis on identity formation in individuals with autism, they argue that this engagement enhances self-awareness, supports meaning-making, and helps to envision one’s future.

Our findings demonstrated a strong engagement in this narrative process, and in line with the suggestion of Samra [53], a positive association was observed between “This is me” statements and improvement in QOL. However, categories within the “I struggle” domain also reflected engagement in a narrative process, but without any positive correlation with QOL. This indicates that different narrative processes may serve different functions, consistent with research arguing that different modes of self-focus have distinct functional properties [54,55]. For example, Watkins et al [54-56] differentiate between maladaptive analytical self-focus—described as abstract, evaluative, and often ruminative—and adaptive experiential self-focus, which is concrete, process-oriented, and linked to positive self-evaluation.

Although we did not deductively categorize statements based on this distinction—maladaptive vs adaptive self-focus—our findings align with the theory and may still be interpreted within this framework, offering potential guidance for clinicians in identifying and supporting beneficial communicative behaviors.

### Limitations

This study design has several limitations. First, only text written via the messaging function of the intervention was included in the analysis; text written directly in response to exercises was excluded, as it was typically constrained by predefined questions and exercises, making it less relevant for the aim of identifying spontaneous communicative behaviors.

It should be noted that the participants included in this study are not fully representative of the entire autistic population, as a natural selection of individuals already familiar with technology applies for inclusion in a trial such as this. Moreover, participants who did not write anything were excluded from the current analyses, which may imply a certain selection bias, as these individuals might have refrained from messaging due to communication difficulties. Those failing to complete the postassessment were also excluded from the correlation analysis, which means that we are unable to conclude the relationship between behavior and outcome for these individuals. However, post hoc analyses with imputed zeros modeling noncorrespondence, as well as imputed zeros for change scores on quantitative outcomes, were conducted, showing that these procedures did not affect the correlational outcomes (data not shown).

Another limitation concerns the role of the primary analyst (BW), who also served as a therapist and was involved in developing the intervention. This dual role introduces a risk of bias, potentially influencing data interpretation, coding, and categorization. To minimize this bias, the initial phase of the analysis was conducted jointly with KJ, who had no previous involvement in treatment development or delivery. Furthermore, the coding approach was deliberately inductive and aimed to be as close to the text as possible. Nevertheless, future studies would benefit from involving fully independent third-party analysts to further strengthen the trustworthiness of the findings.

Another methodological limitation in this study is that the intercoder reliability was not statistically calculated. Typically, around 10%-25% of data units are coded by more than 1 researcher to facilitate a trustworthy estimate of intercoder reliability [57]. By collaborative coding of 5 (14.71%) of the data units in this study, the analysts could compare and discuss codes to develop a preliminary coding framework. By subsequently coding 1 of the participants’ texts independently, the coding framework was further refined and agreed upon. We considered this to be sufficient to establish the final coding framework, but acknowledge that the reliability would be strengthened with additional collaborative coding. Furthermore, in future studies, interrater reliability should be assessed using a statistical test, for example, Cohen κ.

As our analysis did not account for the timing of the statements, it is uncertain whether any detection of potentially beneficial behaviors can be made at an early stage in the therapy. Early detection (or absence) of these behaviors could enable more individualized therapeutic support, such as encouraging these behaviors. Given the novelty of this research area and the fact that similar data have not previously been studied, we chose in this study to focus on broader behavioral patterns rather than their temporal distribution. Future research could consider focusing on early-stage behaviors as potential indicators of treatment response.

When interpreting the results from the correlation analyses, it should be noted that no correction for multiple comparisons was applied. Given the relatively large number of behavioral categories and correlations tested, there is an increased risk of inflated false positives, and the results should be interpreted with caution.

Finally, in the correlational analyses between behaviors and outcome, we did not control for therapist word count or therapist behaviors. Given previous research emphasizing the importance of therapist support and the varying number of therapist messages, therapist behavior may have influenced the relationships. However, since the therapist’s word count was not directly correlated with outcomes in this study, it was not included as a covariate. Future studies should nevertheless consider therapist variables when exploring outcome predictors in ICBT for individuals with autism.

### Conclusions

The findings of this study increase our understanding of how individuals with autism engage in ICBT and may serve as a valuable source of information for therapists in guiding both their expectations of client outcomes and identifying client behaviors to support. The results emphasize the importance of self-narrative formulation in individuals with autism and suggest that certain communicative behaviors—especially those involving identity reflection and recognition of treatment benefits—are positively related to improvements in QOL. Future research should explore ways to identify such behaviors early in treatment as potential indicators of individuals at risk of poorer outcomes, enabling clinicians to provide additional support or tailor interventions to better suit individual needs.

## Appendix

Multimedia Appendix 1 Treatment modules in the internet-based intervention MILAS: overview of domains, categories, definitions, example codes, and quotes from this study and, where applicable, the corresponding categories from previous studies.

## Abbreviations

BBQ: Brunnsviken Brief Quality of Life Scale
HADS: Hospital Anxiety and Depression Scale
HADS-A: Hospital Anxiety and Depression Scale for the anxiety subscale
HADS-D: Hospital Anxiety and depression Scale for the depression subscale
ICBT: internet-based cognitive behavioral therapy
QOL: quality of life
RCT: randomized controlled trial
SOC: sense of coherence
SOC-13: 13-item Sense of Coherence scale

## Data Availability

The data supporting this study originates from a longitudinal intervention study conducted at the University Health Care Research Center, Örebro Region, Sweden. Data are available upon reasonable request from the corresponding author.
