# Supplementary material for: Communicative Behaviors in an Internet-Based Intervention for Individuals With Autism: Mixed Methods Analysis
Source: J Med Internet Res. 2026 Feb 4;28:e76527. doi: 10.2196/76527 (PMC12917485; doi:10.2196/76527)
Supplement: Multimedia Appendix 1 [file jmir_v28i1e76527_app1.docx]

| **Table S1.** Treatment modules in the internet-based intervention MILAS (1), and examples of strategies and exercises in each module. | | |
| --- | --- | --- |
| **Treatment module** | **Examples of strategies involved** | **Exercise** |
| 1. Introduction, diagnostic criteria | - | Reflect on own symptoms and treatment expectations. |
| 2. Behavioural change & individual goals | Setting goals according to the SMART-model | Determine own goal |
| 3. Behavioural change | Functional analyses to understand how thoughts, feelings and behaviours interact | Conduct functional analyses of own behaviour |
| 4. Mentalization | Ask questions, ask for clarification, consult others. | Reflect on misunderstandings, interpret other people’s behaviour |
| 5. Social interaction I – Initiate & keep a conversation going | Active listening, ask questions, associating | Start a conversation |
| 6. Social interaction II – End a conversation, talking by phone | Use script in conversations, using of white lies when suitable | Practice phone calls |
| 7. Problem solving | Problem solving in 6 steps | Try a problem solving technique |
| 8. Emotions & non-verbal communication | Interpreting body language and nonverbal signals | Observe body language in a discussion |
| 9. Perception | Usage of sun glasses, ear buds, headphones, weight blanket, informing others of preferences | Reflect on own perceptual deviances |
| 10. Depression | Behavioural activation: identify and plan positive activities | Plan and carry through positively reinforced activities |
| 11. Social anxiety | Exposure, identify and challenge safety behaviours | Exposure to a social situation. |
| 12. Central coherence & logical errors | Question the plausibility of thoughts, consulting others | Reflect on experiences of logical errors and detail focus. Cognitive restructuring. |
| 13. Relations | Show appreciation, setting boundaries, honesty and trust | Reflect on emotions, friendship and love. Show appreciation to a loved one |
| 14. Stress and sleep | Specify what give and takes energy. Develop structure and routines around sleep, relaxation exercises | Plan and implement relaxation exercises |
| 15. Organization at home | Several strategies regarding planning and structure. | Try a new strategy to facilitate everyday activities |
| 16. Employment | Awareness of talents, personal traits and difficulties | Reflect on personal traits. Keep a journal of food intake and exercise (For next module) |
| 17. Diet & physical exercise | The plate model, regular meals | Evaluate the journal of food and exercise, and decide on something to change |
| 18. Summary | - | Evaluation |

**Table S2.** Overview of domains, categories, definitions, example codes, and quotes from each category

| Domain and category | | Definition | Example codes | Example quote |
| --- | --- | --- | --- | --- |
| **This is me** | | | | |
|  | This is who I am | Texts reflecting a personal narrative about what kind of person one is, what qualities, difficulties, and needs they have, their autistic functioning, and how they (think that they) are perceived by others. | I think a lot in pictures.  I have never been a bookworm.  People have a hard time reading me. | “I keep jumping back and forth in the conversation to compare what was said now and the current facial expressions, tone of voice, etc., with what was said a few seconds ago to double-check that I interpreted it correctly.” |
|  | My present and past circumstances | Information on current events, everyday life, positive experiences, and early formative events. | I had a lot of support in school.  The new job is at a […] school.  I have good people in my life. | “In high school, I finally made friends! Unfortunately, I couldn’t focus as well on my studies while maintaining friendships. […] After high school, I spent many years with temporary jobs and a few university courses.” |
|  | Change is possible | Strategies that work or have potential to work, that they have developed through life, and show motivation and insight that they have the agency to act to progress. | Knitting works for anxiety.  I've quit with charades.  I never give up. | “I’m aware that the connection between my facial muscles and emotions is loose, but I’ve chosen to accept it and, if the situation requires, inform the other party about how I (don’t) function.” |
|  | My point of view | Beliefs, opinions, and thoughts about society, autism, human psychology, and the perspective of others. | I miss depth in the debate today.  She might have done that because…  It’s different how one views getting a diagnosis. | “Most people don’t know much about autism, and even fewer have experience accommodating the differences of autistic people. That’s why they can’t put themselves in my position enough to understand the difficulties I face.” |
| **Working with the treatment** | | | | |
|  | Appreciation and treatment alliance | Expressions of appreciation, a positive attitude, and bonds of alliance toward the treatment or the therapist. | Thank you for the great feedback!  I liked the problem-solving strategy.  This is going to be exciting! | “I want to take the opportunity to thank you for the way you highlight the positive aspects of me. You’ve given me a lot to think about and some help with various challenges.” |
|  | Putting the treatment aside | Information about not having completed parts of the treatment, and reasons for this, including both psychological issues, such as forgetting and lacking energy, but also external events that got in the way of the treatment work. | I missed it because of forgetfulness.  I haven’t responded because I got sick.  I've procrastinated on the task. | “I have (as usual) completely lost my motivation once I’ve gotten a bit into something.” |
|  | Plans to attempt a new task | Reflections regarding how to – or plans to implement a treatment task or exercise, or deciding on a treatment goal. | I will start with the module today.  Rumination is something long-term to work on.  I’m going to get storage boxes to sort things. | “I think I’ll try using an activity journal. I'll start with it today.” |
|  | Have attempted a new task | Reports on completion of a treatment task or exercise, or reflections around the implementation of a task or exercise. | I did the home assignment today.  I chatted with a new colleague.  Now I’m done with module 8. | “I found it interesting to observe body language. I didn't notice anything surprising.” |
|  | Observing positive consequences of treatment | Statements illustrating that the participant has observed a positive consequence on their personal development from the treatment or specific exercises. | Now I stop myself and live in the moment.  This has given me insight into myself.  The exercise became a kick in the butt. | “Even the small things, like going out and challenging myself and noticing that others do the same, have given me more space to ‘fail’ with myself. It has simply given me insight into myself and others, which makes it easier to mentally move forward.” |
|  | Problems with the treatment | Texts in which the participant expresses difficulties, frustration, or other negative aspects of the treatment content or format, such as failure to understand a task or its purpose, considering certain parts of the treatment irrelevant, or technical problems. | The relaxation exercise was difficult.  It became stressful with your reminders.  I already know how to small-talk, so exercise is irrelevant. | “I probably need to say that I got so frustrated with the exercise. It felt like I was being forced to converse in a way that doesn’t feel good or natural to me.” |
| **I struggle** | | | | |
|  | Life is and has been demanding | Descriptions of current and past difficult events or circumstances, that they have been treated badly throughout life, experienced failure, and have been enduring from mental ill-health. | I’m fighting with the Social Insurance Agency.  It’s really a lot right now.  After all those years of bullying. | “My work managers have belittled me, saying things like, ‘Do you understand anything at all?’ and ‘The kid doesn’t get it.’” |
|  | Identifies patterns and problem behaviors | Texts reflecting identification and insight into maladaptive and safety behaviors, and identification of the relation between these behaviors and negative consequences. | I’ve built up a mask my whole life.  I clasp my hands as a safety thing.  The loneliness is due to my cutting ties too much. | “Every time something goes wrong, and there’s a misunderstanding, I close myself off and refuse to talk about things like feelings, because if I don’t talk about them, others can't misunderstand, and I avoid the anxiety of it going wrong and being a burden.” |
|  | I am troubled by mental ill-health | Factual descriptions of current mental ill-health, loneliness, and struggles and their causes and consequences, without engaging in maladaptive thoughts or rumination. | I push myself to be able to live.  I have a tendency to ruminate.  I have a lot of anxiety. | “It’s due to anxiety, and sometimes I just want to scream and hit the wall. I think I’m prone to anxiety and panic attacks in certain situations because of everything that has happened.” |
|  | Maladaptive thoughts | Expressions of hopelessness, meaninglessness, or distress, as well as other cognitive distortions, indicate a stagnation of cognitive flexibility. | The days feel unbearable.  Everyone thinks I'm the problem.  I'm leaking like a sunken boat. | “I know that people close to me will betray and hurt me when the chance arises; it’s not something I need to think about, it’s just how it's always been.” |

| **Table S3**. Domains and categories from the current study and – where applicable – the corresponding category from the studies of Svartvatten, 2015 (2) and Kraepelien, 2021 (3) | | | |
| --- | --- | --- | --- |
| Domain | Category | Corresponding category in previous studies | Definition in previous studies |
| **This is me** | This is who I am | N/A^a^ | N/A |
|  | My present and past circumstances | N/A | N/A |
|  | Change is possible | N/A | N/A |
|  | My point of view | N/A | N/A |
| **Working with the treatment** | Appreciation and treatment alliance | Alliance | Text expressing the participant's emotional ties in relation to treatment goals, the exercises or the internet therapist |
|  | Putting the treatment aside | Avoidance of treatment | Text about the participant not having completed various parts of the treatment, or not having been as active with the treatment as anticipated, from either a technical or a content-related aspect. |
|  | Plans to attempt a new task | Chooses alternative behavior (Svartvatten).  Plans to attempt behavior change or use treatment exercise (Kraepelien). | Text regarding thoughts about, or plans regarding,implementing a future adaptive behavior or treatment exercise. |
|  | Have attempted a new task | Tries alternative behavior (Svartvatten).  Reports on behavior change attempt or use of treatment exercise (Kraepelien). | Text that demonstrates that the participant has completed, or attempted to complete, a specific alternative behavior or treatment exercise |
|  | Observing positive consequences of treatment | Observes positive consequences of behavior change attempt or use of treatment exercise. | Text that expresses a positive change after the participant tried a specific alternative behavior or exercise in the treatment |
|  | Problems with the treatment | Problems with treatment content | Text that expresses difficulties in filling out, reading or understanding the content or purpose of the treatment material. |
|  |  | Problems with techniques and administration | Text in which the participant expresses difficulties and problems with techniques and administration relating to the platform, such as having problems logging in or understanding the platform |
| **I struggle** | Life is and has been demanding | N/A | N/A |
|  | Identifies patterns and problem behaviors | Identifies patterns and problem behaviors | Text in which the participant identifies the relationship between internal and external behaviors and their effect on the participant's affective condition, alternatively, text that identifies avoidance and rumination. |
|  | I am troubled by mental ill-health | Maladaptive repetitive thinking (Svartvatten)  Maladaptive thinking or anticipation of failure (Kraepelien) | Text about the participant's depressive symptoms and their consequences without any suggested solution, where the future is described as bleak or where the participant anticipates future failure. |
|  | Maladaptive thoughts |  |  |

^a^ Not applicable

## References

1. Westerberg B, Holländare F, Bejerot S. An internet-based behavioral intervention for adults with autism spectrum disorder – A randomized controlled trial and feasibility study. Internet Interv. 2023;34.

2. Svartvatten N, Segerlund M, Dennhag I, Andersson G, Carlbring P. A content analysis of client e-mails in guided internet-based cognitive behavior therapy for depression. Internet Interv. 2015;2(2):121–7.

3. Kraepelien M, Hadjistavropoulos HD, Berman AH, Sundström C. Exploring client messages in a therapist-guided internet intervention for alcohol use disorders – A content analysis. Internet Interv. 2021 Dec;26:100483.
